# Stage-specific integrated nursing management enables successful transition to left ventricular assist device support in end-stage dilated cardiomyopathy: a case report

**DOI:** 10.3389/fcvm.2026.1935797

**Published:** 2026-10-27

**Authors:** Jiawei Wu, Xinrui Chang, Tingting Lang, Zhen Yu, Chan Zhang, Jicheng Xi, Baosheng Han

**Affiliations:** 1 Department of Surgical Intensive Care Unit, Shanxi Cardiovascular Hospital, Taiyuan, China; 2 Shanxi Key Laboratory of Heart Failure Precision Medicine, Taiyuan, China; 3 Department of Cardiovascular Surgery, Shanxi Cardiovascular Hospital, Taiyuan, China

**Keywords:** dilated cardiomyopathy, end-stage heart failure, extracorporeal membrane oxygenation, integrated nursing management, left ventricular assist device

## Abstract

**Introduction:**

Patients with end-stage heart failure complicated by cardiogenic shock and multiple organ dysfunction often require sequential mechanical circulatory support. However, evidence regarding integrated nursing management during transitions between multiple life-support modalities remains limited. This case report describes the nursing management of a patient with end-stage dilated cardiomyopathy who successfully transitioned from acute rescue therapy to long-term mechanical circulatory support.

**Case presentation:**

A 39-year-old female with end-stage dilated cardiomyopathy complicated by cardiogenic shock and subsequent cardiac arrest underwent sequential life support interventions, including an intra-aortic balloon pump, extracorporeal membrane oxygenation, continuous renal replacement therapy, and ultimately, left ventricular assist device implantation. A dynamic, phase-based integrated nursing management strategy was implemented across four distinct clinical stages, focusing on precise hemodynamic perfusion monitoring, coordinated fluid and electrolyte balance during combined extracorporeal therapies, perioperative infection prevention, and early multi-dimensional physical and psychological rehabilitation. Following this individualized, multidisciplinary nursing approach, the patient's hemodynamic status stabilized, renal and respiratory functions recovered, and she was successfully discharged with favorable functional independence.

**Conclusion:**

Stage-specific integrated nursing management represents a vital conceptual advance in critical care, serving as a central coordinating mechanism that optimizes treatment continuity, enhances organ recovery, and ensures a safe transition to long-term survival in patients facing refractory circulatory failure.

## Introduction

Advanced heart failure (AHF) represents the terminal stage of cardiovascular disease and is characterized by persistent low cardiac output, inadequate tissue perfusion, and progressive multiorgan dysfunction, resulting in substantial morbidity and mortality. Dilated cardiomyopathy (DCM) is a common cause of end-stage heart failure, and some patients deteriorate rapidly despite guideline-directed medical therapy, progressing to cardiogenic shock and requiring mechanical circulatory support (1).

Extracorporeal membrane oxygenation (ECMO) can provide rapid temporary circulatory and respiratory support in patients with refractory cardiogenic shock, serving as a bridge to recovery or further intervention. However, prolonged ECMO support is associated with significant risks, including bleeding, thrombosis, infection, and hemodynamic instability (1). Acute kidney injury frequently develops during severe circulatory failure and ECMO support, making continuous renal replacement therapy (CRRT) an important adjunct for fluid management, metabolic regulation, and organ support (2, 3).

For patients with irreversible cardiac dysfunction, left ventricular assist devices (LVADs) provide durable mechanical circulatory support as either a bridge to transplantation or destination therapy (4). In clinical practice, critically ill patients may require sequential support with intra-aortic balloon pump (IABP), ECMO, CRRT, and ultimately LVAD implantation. Successful transition through these treatment stages requires not only timely clinical decision-making but also meticulous multidisciplinary care.

Nursing management is particularly challenging during multimodal mechanical circulatory support because patient status can change rapidly and complications may arise at every stage of treatment. Continuous hemodynamic assessment, anticoagulation monitoring, infection prevention, fluid regulation, nutritional support, and early rehabilitation are all essential to achieving favorable outcomes. Despite the increasing use of advanced mechanical circulatory support, reports describing integrated nursing management throughout the transition from temporary life support to durable ventricular assist device therapy remain scarce.

Here, we report a patient with end-stage DCM complicated by cardiogenic shock, cardiac arrest, acute kidney injury, and multiorgan dysfunction who successfully transitioned from IABP support and ECMO–continuous renal replacement therapy to LVAD implantation. This case highlights the critical role of stage-specific integrated nursing management in facilitating organ recovery, preventing complications, and supporting successful transition to long-term mechanical circulatory support.

## Case presentation

### Patient information and clinical findings

A 39-year-old woman with a history of DCM and chronic heart failure was admitted to the intensive care unit because of progressive fatigue, worsening circulatory insufficiency, and signs of low cardiac output. Despite ongoing medical therapy for heart failure, her condition had progressively deteriorated before admission. On presentation, she was in cardiogenic shock with severe hemodynamic instability. Her blood pressure was 88/66 mmHg, and serum lactate was markedly elevated at 14 mmol/L, indicating profound tissue hypoperfusion. Clinical evaluation suggested AHF with impending multiorgan dysfunction. The overall clinical timeline and the perfusion-assessment framework used during ECMO support are presented in Figures 1, 2, respectively.

**Figure 1:**
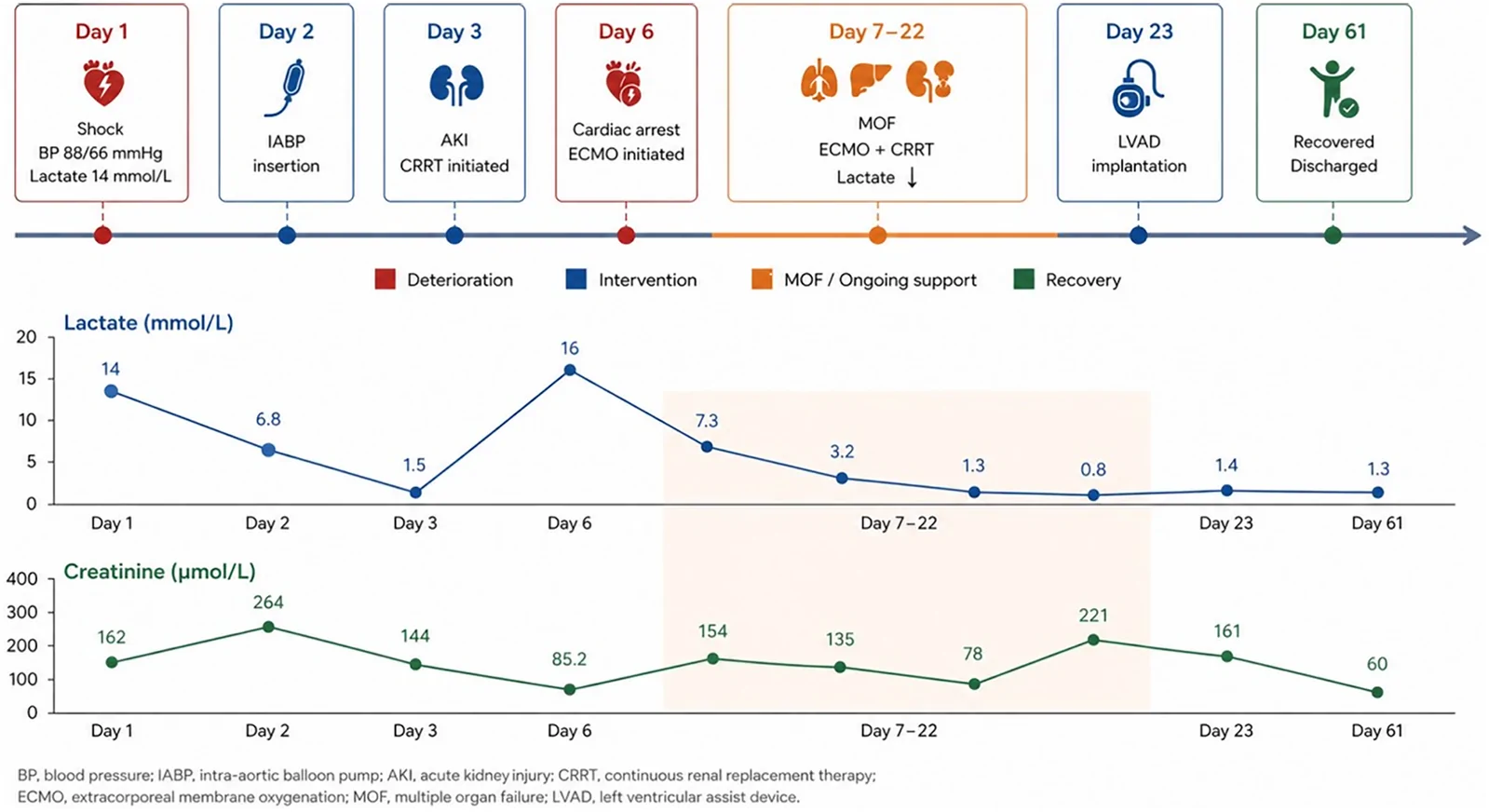
Clinical timeline and dynamic changes in key clinical indicators during multimodal mechanical circulatory support. The patient's clinical course from admission to discharge, including IABP insertion, initiation of CRRT, extracorporeal cardiopulmonary resuscitation with ECMO, combined ECMO–CRRT support, and LVAD implantation. Serial serum lactate and creatinine levels demonstrate progressive improvement in tissue perfusion and renal function during treatment. BP, blood pressure; IABP, intra-aortic balloon pump; AKI, acute kidney injury; CRRT, continuous renal replacement therapy; ECMO, extracorporeal membrane oxygenation; MOF, multiple organ failure; LVAD, left ventricular assist device.

**Figure 2:**
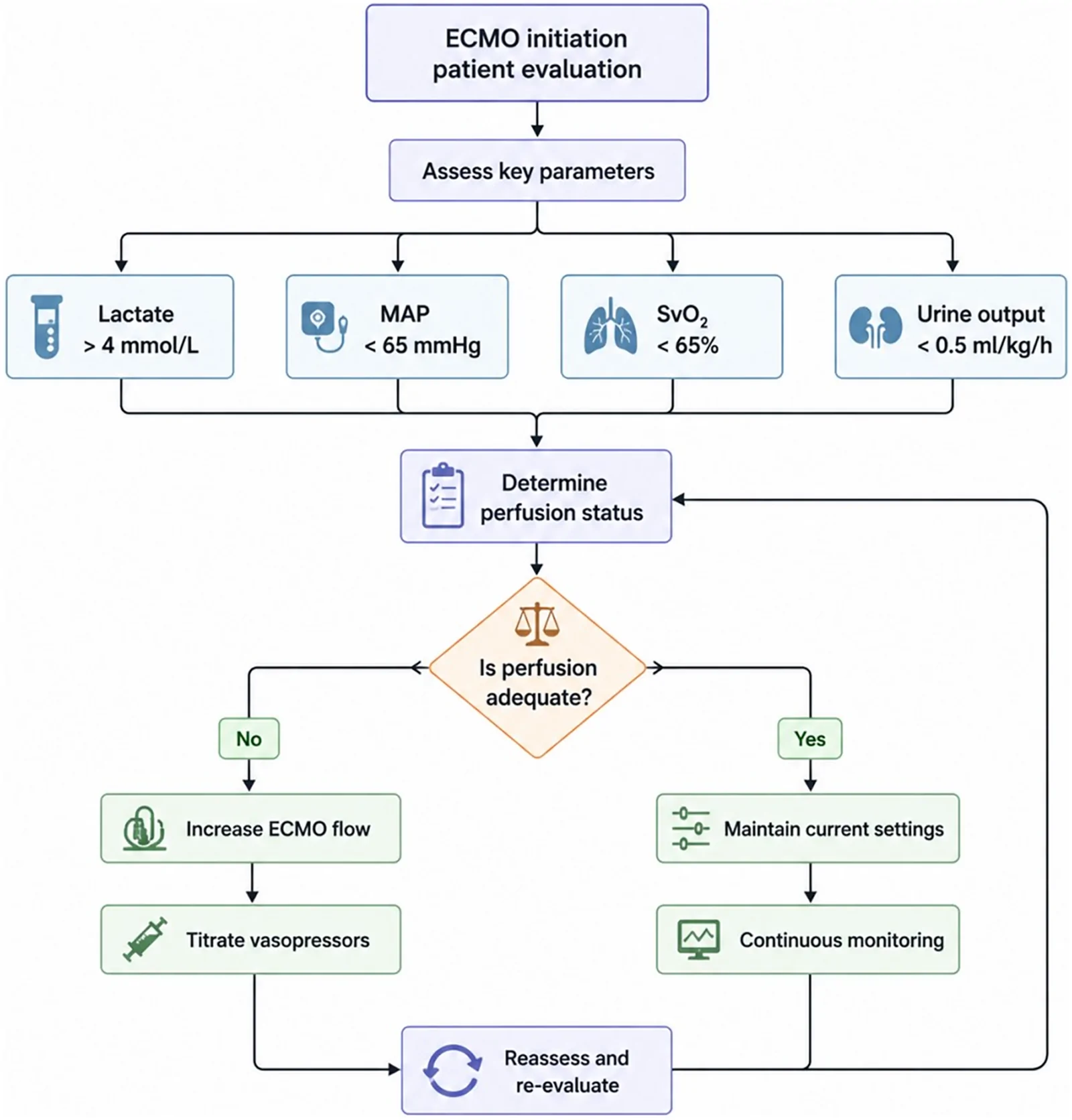
Hemodynamic assessment and perfusion management during ECMO support. Algorithm for dynamic assessment of tissue perfusion during ECMO support. Perfusion status was evaluated using serum lactate levels, MAP, SvO_2_, and urine output. ECMO flow and vasoactive therapy were adjusted according to these parameters to maintain adequate systemic perfusion and hemodynamic stability. MAP, mean arterial pressure; SvO_2_, mixed venous oxygen saturation; ECMO, extracorporeal membrane oxygenation.

### Diagnostic assessment

The diagnosis of end-stage heart failure secondary to DCM complicated by cardiogenic shock was established based on the patient's medical history, clinical manifestations, laboratory findings, and imaging studies. Following admission, serial laboratory testing demonstrated progressive renal dysfunction, oliguria, hepatic impairment, and systemic inflammatory responses, indicating evolving multiple organ dysfunction. Chest radiography revealed severe pulmonary congestion before initiation of extracorporeal support (Figure 3A). Echocardiography demonstrated severe left ventricular systolic dysfunction, consistent with advanced DCM (Figure 4A). During hospitalization, the patient developed malignant arrhythmia that progressed to cardiac arrest. Given the underlying end-stage cardiomyopathy, severe circulatory collapse, and absence of alternative causes of shock, the clinical diagnosis remained cardiogenic shock complicated by acute kidney injury and multiple organ dysfunction syndrome.

**Figure 3:**
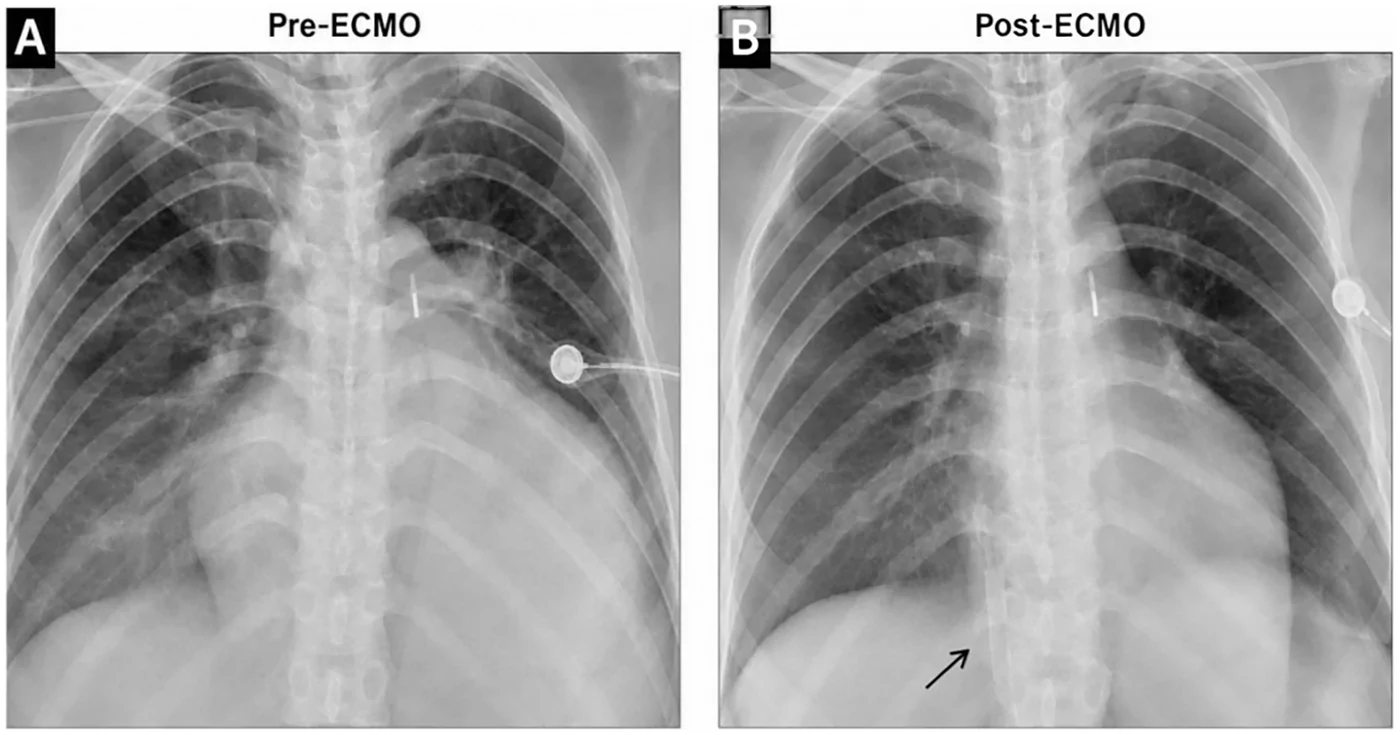
Chest radiographic findings before and after ECMO support. **(A)** Chest radiograph obtained before ECMO initiation showing cardiomegaly with diffuse pulmonary congestion and pulmonary edema. **(B)** Follow-up chest radiograph after ECMO support demonstrating marked resolution of pulmonary congestion and pulmonary edema. The arrow indicates the tip of the ECMO cannula.

**Figure 4:**
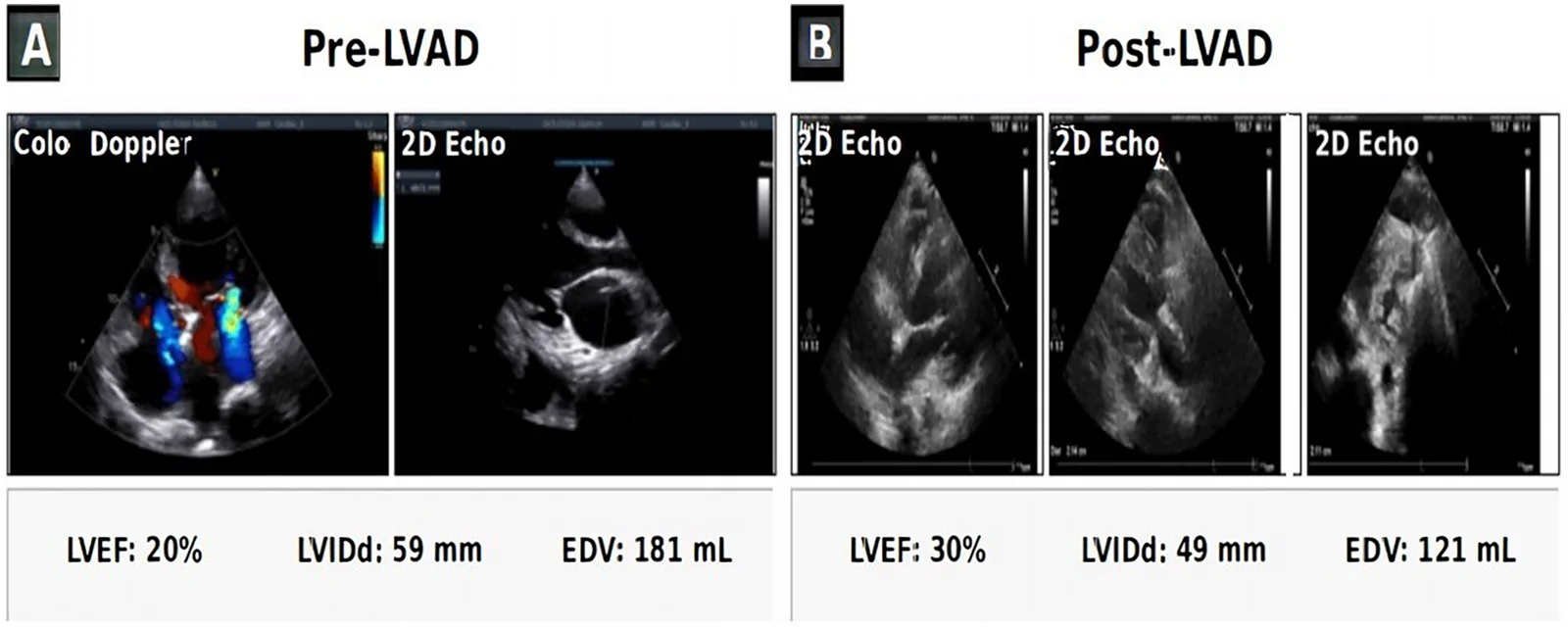
Echocardiographic findings before and after LVAD implantation. **(A)** Preoperative echocardiography demonstrating severe left ventricular systolic dysfunction, with a LVEF of 20%, LVIDd of 59 mm, and EDV of 181 mL. **(B)** Postoperative echocardiography demonstrating improved left ventricular systolic function following LVAD implantation, with an LVEF of 30%, LVIDd of 49 mm, and EDV of 121 mL, indicating effective ventricular unloading and reverse remodeling. LVAD, left ventricular assist device; LVEF, left ventricular ejection fraction; LVIDd, left ventricular internal diameter in diastole; EDV, end-diastolic volume.

### Timeline and therapeutic intervention

The major clinical events and therapeutic interventions are summarized in Figure 1. Initial management focused on stabilization of cardiogenic shock with vasoactive agents and IABP support. However, despite aggressive treatment, her hemodynamic status continued to deteriorate. Following cardiac arrest, cardiopulmonary resuscitation was immediately initiated, and extracorporeal cardiopulmonary resuscitation with venoarterial ECMO (VA-ECMO) was established to provide circulatory and respiratory support. After ECMO initiation, systemic perfusion and oxygenation improved. Nevertheless, acute kidney injury, fluid overload, and persistent multiorgan dysfunction developed during the course of treatment. CRRT was therefore initiated to optimize fluid balance, maintain electrolyte and acid–base homeostasis, and support organ recovery. With ongoing multidisciplinary management, serum lactate levels gradually decreased, renal function improved, and inflammatory markers declined. After recovery of tissue perfusion and stabilization of organ function, the patient was deemed suitable for long-term mechanical circulatory support and successfully underwent LVAD implantation. This marked the transition from temporary rescue therapy to durable circulatory support.

### Follow-up and outcomes

Following LVAD implantation, the patient's hemodynamic status progressively stabilized. ECMO and IABP support were successfully withdrawn, and no major procedure-related complications occurred during the transition period. Serial laboratory assessments demonstrated gradual normalization of serum lactate levels, improvement in renal function, recovery of hepatic function, and reduction of inflammatory markers. Chest radiography showed marked resolution of pulmonary congestion compared with pre-ECMO findings (Figure 3B). Postoperative echocardiography demonstrated improved cardiac performance and reduced ventricular dilation following LVAD support (Figure 4B). The patient tolerated all therapeutic interventions well and experienced no severe catheter-related infections, major bleeding events, or other unexpected adverse events during hospitalization. Following comprehensive rehabilitation and recovery, she was discharged in stable condition with satisfactory clinical improvement.

### Nursing management

Nursing management was tailored to the patient's evolving clinical condition and implemented throughout the course of multimodal mechanical circulatory support. According to the major therapeutic stages, nursing care focused on four key areas: hemodynamic management during ECMO support, coordinated management during combined ECMO and CRRT, perioperative management during transition to LVAD support, and postoperative rehabilitation after LVAD implantation.

### Hemodynamic management during ECMO support

Following initiation of venoarterial ECMO, the primary nursing objective was restoration of adequate tissue perfusion and maintenance of hemodynamic stability. As illustrated in Figure 2, tissue perfusion was assessed continuously using a combination of serum lactate levels, mean arterial pressure, mixed venous oxygen saturation, urine output, and arterial blood gas analysis. These parameters were evaluated dynamically to determine whether systemic oxygen delivery was sufficient to meet metabolic demands. Particular attention was paid to lactate clearance as an indicator of circulatory recovery. When signs of inadequate perfusion were identified, including persistent hyperlactatemia, reduced urine output, or declining arterial pressure, the nursing team promptly communicated these findings to the multidisciplinary team and assisted with adjustment of ECMO flow settings and vasoactive medications. Fluid status was assessed through continuous monitoring of central venous pressure, urine output, bedside ultrasonography, and pulmonary imaging findings. The nursing goal was to maintain adequate preload while avoiding fluid overload, which could worsen pulmonary edema and increase cardiac workload. Through continuous reassessment and individualized volume management, tissue perfusion and hemodynamic stability were progressively restored.

### Coordinated management during combined ECMO and CRRT support

As acute kidney injury and fluid overload developed during ECMO support, CRRT was initiated to provide multiorgan support. Supplementary Figure S1 summarizes the coordinated management strategy used during this phase. Nursing care focused on maintaining fluid balance, correcting electrolyte disturbances, supporting renal recovery, and minimizing treatment-related complications. Strict intake and output monitoring was performed, and ultrafiltration targets were adjusted according to hemodynamic parameters, urine output, and central venous pressure measurements. Continuous fluid removal through CRRT facilitated precise volume control while avoiding excessive preload reduction that could compromise systemic perfusion. Electrolyte concentrations, acid–base status, and metabolic parameters were monitored through serial laboratory testing and arterial blood gas analysis. Abnormalities, particularly hyperkalemia and metabolic acidosis, were identified promptly and corrected through adjustment of CRRT settings and replacement fluids. Anticoagulation management represented another critical component of care. Coagulation parameters were monitored regularly to guide anticoagulant therapy and balance the competing risks of thrombosis and bleeding. The nursing team also assessed filter pressures and circuit performance to identify early signs of filter clotting or circuit dysfunction and ensure uninterrupted CRRT delivery. Infection prevention measures were implemented throughout this period. Catheter insertion sites were inspected regularly, strict aseptic techniques were maintained, and body temperature, white blood cell count, and inflammatory markers were monitored closely to facilitate early detection of infectious complications.

### Transitional management from ECMO to LVAD support

Following gradual recovery of tissue perfusion and organ function, nursing priorities shifted toward preparation for LVAD implantation. As shown in Supplementary Figure S2, transition to durable mechanical circulatory support required comprehensive multidisciplinary assessment of coagulation status, infection control, and end-organ recovery. The nursing team performed continuous monitoring of renal and hepatic function, serum lactate levels, inflammatory markers, and hemodynamic parameters to evaluate readiness for surgery. Fluid management strategies were optimized to improve perioperative stability and reduce surgical risk. Given the prolonged duration of invasive support, infection surveillance remained a major priority. Daily assessment of body temperature, laboratory inflammatory markers, and catheter sites was performed, while strict infection-control measures were maintained to minimize perioperative infectious complications. Coagulation monitoring was intensified during the transition period because anticoagulation requirements differ between ECMO and LVAD support. Activated clotting time, activated partial thromboplastin time, international normalized ratio, and platelet counts were assessed regularly, and anticoagulation regimens were adjusted collaboratively to reduce the risks of thrombosis and perioperative bleeding. In addition, neurological status, respiratory function, and overall treatment tolerance were assessed continuously. The nursing team coordinated preoperative preparation and assisted in ensuring a safe transition from temporary extracorporeal support to durable ventricular assist device therapy.

### Postoperative rehabilitation following LVAD implantation

After LVAD implantation, nursing care shifted from life-sustaining support toward functional recovery and long-term rehabilitation. A staged rehabilitation pathway was implemented, as outlined in Supplementary Figure S3. Early rehabilitation began with passive range-of-motion exercises, bed mobility training, and position management to prevent pressure injuries, venous thromboembolism, and muscle deconditioning. As the patient's condition stabilized, rehabilitation progressed gradually from sitting at the bedside to standing and ambulation training. Respiratory rehabilitation was integrated throughout recovery. Deep-breathing exercises, effective coughing techniques, and incentive spirometry were encouraged to improve pulmonary ventilation and reduce the risk of atelectasis and pulmonary infection. Nutritional management was individualized according to the patient's metabolic requirements. Nutritional status was monitored through serial assessment of body weight, serum albumin levels, and overall dietary intake to support wound healing and functional recovery. Psychological support was provided throughout hospitalization. Following cardiac arrest, prolonged mechanical circulatory support, and major cardiac surgery, the patient experienced substantial emotional stress. Ongoing communication, education, and counseling were therefore incorporated into routine care to enhance treatment adherence, participation in rehabilitation, and confidence in long-term self-management. Through this stage-specific nursing strategy, the patient successfully progressed from critical illness to functional recovery, ultimately achieving stable discharge after successful LVAD implantation.

## Discussion

This case describes the successful management of a patient with end-stage DCM complicated by cardiogenic shock, cardiac arrest, acute kidney injury, and multiple organ dysfunction. Through a staged multimodal mechanical circulatory support strategy and integrated nursing management, the patient successfully transitioned from acute rescue therapy to durable LVAD support and ultimately achieved favorable recovery. The case highlights not only the value of sequential mechanical circulatory support but also the critical role of nursing care throughout the transition process.

### Multimodal mechanical circulatory support as a bridging strategy

Patients with end-stage heart failure who develop refractory cardiogenic shock frequently require temporary mechanical circulatory support to restore systemic perfusion and preserve end-organ function. ECMO has become an established rescue therapy in this setting because it can rapidly provide circulatory and respiratory support during severe hemodynamic collapse (1, 5). However, ECMO alone does not address irreversible myocardial dysfunction and is associated with substantial risks when prolonged support is required (1, 5).

In the present case, ECMO served as an effective bridge following cardiac arrest and extracorporeal cardiopulmonary resuscitation, allowing restoration of tissue perfusion and stabilization of vital organ function. However, acute kidney injury and fluid overload developed during the course of treatment. CRRT was therefore incorporated to facilitate precise fluid management, metabolic control, and maintenance of internal homeostasis. Previous studies have shown that combined ECMO–CRRT support may improve management of critically ill patients with multiorgan dysfunction by addressing both circulatory and renal failure simultaneously (3). As end-organ function gradually recovered, the patient was successfully transitioned to LVAD support. This staged progression from temporary rescue therapy to durable circulatory support reflects an increasingly recognized strategy for managing AHF (4). The favorable outcome observed in this case underscores the importance of continuous reassessment of hemodynamic status, organ recovery, and treatment tolerance when determining the optimal timing for transition between support modalities.

### Importance of integrated nursing management

Although mechanical circulatory support technologies provide the physiological foundation for recovery, successful treatment depends heavily on effective nursing management. The complexity of caring for patients receiving ECMO, CRRT, and LVAD support requires continuous monitoring, rapid clinical assessment, and coordinated multidisciplinary communication. During ECMO support, nursing priorities focused on early identification of inadequate tissue perfusion through dynamic monitoring of serum lactate levels, arterial pressure, urine output, and oxygenation parameters. During combined ECMO–CRRT therapy, nursing management expanded to include individualized fluid regulation, electrolyte and acid–base management, anticoagulation monitoring, and surveillance for circuit-related complications. As the patient approached LVAD implantation, attention shifted toward assessment of organ recovery, infection prevention, perioperative preparation, and coagulation management. Following surgery, rehabilitation-centered interventions, including progressive mobilization, respiratory training, nutritional support, and psychological counseling, facilitated functional recovery and improved treatment adherence. Previous studies have demonstrated the benefits of standardized infection-control measures and multidisciplinary care pathways in critically ill patients receiving advanced life support (6, 7). However, most published reports focus on isolated nursing interventions or single support modalities. In contrast, the present case illustrates a stage-specific nursing strategy that evolved in parallel with the patient's changing physiological needs. This approach enabled seamless coordination across different treatment phases and may have contributed substantially to the successful clinical outcome.

### Clinical implications

Several clinically relevant observations emerge from this case. First, successful recovery was achieved through the timely integration of multiple support modalities rather than reliance on any single intervention. Second, dynamic assessment of organ function and treatment response was essential for determining when to escalate or transition support. Third, nursing management functioned as a continuous coordinating framework throughout the treatment course, linking acute resuscitation, multiorgan support, perioperative care, and rehabilitation. The favorable outcome observed in this patient likely resulted from the combined effects of early circulatory stabilization, meticulous organ-support strategies, prevention of treatment-related complications, and coordinated multidisciplinary care. Although causality cannot be established in a single case report, this experience suggests that structured nursing management may play an important role in maximizing the benefits of advanced mechanical circulatory support.

### Strengths and limitations

A major strength of this report is the comprehensive description of nursing management throughout the entire trajectory of multimodal mechanical circulatory support, from ECMO initiation and CRRT integration to LVAD implantation and postoperative rehabilitation. The inclusion of structured management pathways and patient-specific clinical outcomes provides practical guidance for clinicians and nurses caring for similarly complex patients.

Several limitations should also be acknowledged. First, this report describes a single patient, limiting the generalizability of the findings. Second, some physiological and nursing-related indicators were not collected prospectively or analyzed quantitatively throughout all treatment stages. Third, the optimal timing for initiation of multimodal support, transition between support modalities, and implementation of standardized nursing pathways remains uncertain and requires validation in larger multicenter studies. Finally, because of the observational nature of a case report, the independent contribution of individual nursing interventions cannot be determined.

## Conclusion

This case demonstrates that staged multimodal mechanical circulatory support, combined with integrated nursing management, can facilitate successful recovery in patients with end-stage DCM complicated by cardiogenic shock and multiorgan dysfunction. Continuous assessment of tissue perfusion, meticulous fluid and anticoagulation management, prevention of treatment-related complications, and structured rehabilitation were key components of care throughout the treatment course. The experience highlights the importance of dynamic multidisciplinary collaboration and suggests that stage-specific nursing management may be an essential element in supporting the transition from temporary life-saving interventions to durable mechanical circulatory support and long-term recovery.

## Data Availability

The original contributions presented in the study are included in the article/Supplementary Material, further inquiries can be directed to the corresponding author.
