# Supplementary material for: Stage-specific integrated nursing management enables successful transition to left ventricular assist device support in end-stage dilated cardiomyopathy: a case report
Source: Front Cardiovasc Med. 2026 Oct 27;13:1935797. doi: 10.3389/fcvm.2026.1935797 (PMC13747964; doi:10.3389/fcvm.2026.1935797)
Supplement: Supplementary file 1 [file Datasheet1.docx]

**Supplementary Fig. 1. Coordinated management during combined ECMO and CRRT support**

Schematic representation of acute kidney injury development during ECMO support and the coordinated management strategy implemented during combined ECMO–CRRT therapy. Key interventions included individualized volume management, electrolyte and acid–base regulation, and control of systemic inflammation to promote cardiorenal recovery and maintain internal homeostasis.

Abbreviations: ECMO, extracorporeal membrane oxygenation; CRRT, continuous renal replacement therapy; AKI, acute kidney injury; CVP, central venous pressure; CRP, C-reactive protein.

**Supplementary Fig. 2. Multidisciplinary assessment and transition from ECMO to LVAD support**

Framework for evaluating readiness for LVAD implantation during ECMO support. Transition criteria included stable coagulation status, adequate infection control, and recovery of end-organ function. After meeting these criteria, LVAD implantation was performed, followed by successful weaning and discontinuation of ECMO support.

Abbreviations: ECMO, extracorporeal membrane oxygenation; LVAD, left ventricular assist device; CRRT, continuous renal replacement therapy; ACT, activated clotting time; INR, international normalized ratio; CRP, C-reactive protein.

**Supplementary Fig.3. Staged rehabilitation pathway following LVAD implantation.**

Structured rehabilitation program after LVAD implantation, including early mobilization, progressive functional training, cardiopulmonary rehabilitation, nutritional support, and psychological care. The rehabilitation pathway aimed to restore functional capacity, facilitate recovery, and improve long-term quality of life.

Abbreviations: LVAD, left ventricular assist device.
